# Epstein-Barr virus infection and clinical outcome in breast cancer patients correlate with immune cell TNF-α/IFN-γ response

**DOI:** 10.1186/1471-2407-14-665

**Published:** 2014-09-11

**Authors:** Gina Marrão, Mohammed Habib, Artur Paiva, Dominique Bicout, Catherine Fallecker, Sofia Franco, Samira Fafi-Kremer, Teresa Simões da Silva, Patrice Morand, Carlos Freire de Oliveira, Emmanuel Drouet

**Affiliations:** Université de Grenoble-Alpes, Unit for Virus Host-Cell Interactions, UMI 3265 UJF-CNRS-EMBL, CIBB, 71 Avenue des Martyrs, F-38042 Grenoble, Cedex 9, France; Portuguese Institute for Blood and Transplantation, University Hospital, Coimbra, Portugal; Team Environment and Health Prediction in Populations Unit – TIMC Laboratory, UMR CNRS 5525, Université Joseph Fourier, Grenoble, France; Department of Gynecology, University Hospital, Coimbra, & Faculty of Medicine, University of Coimbra, Coimbra, Portugal; Unit of Virology, University Hospital, Grenoble, France; Department of Pathology, University Hospital, Coimbra, Portugal; Laboratoire de Virologie, Hôpitaux Universitaires de Strasbourg, Université de Strasbourg, Strasbourg, France

**Keywords:** Breast cancer, EBV, Viral load, Tumor, Immunocompetent cells, IFN-γ, TNF-α, Survival, Multivariate analysis, ZEBRA

## Abstract

**Background:**

For nearly two decades now, various studies have reported detecting the Epstein-Barr virus (EBV) in breast cancer (BC) cases. Yet the results are unconvincing, and their interpretation has remained a matter of debate. We have now presented prospective data on the effect of EBV infection combined with survival in patients enrolled in a prospective study.

**Methods:**

We assessed 85 BC patients over an 87-month follow-up period to determine whether EBV infection, evaluated by qPCR in both peripheral blood mononuclear cells (PBMCs) and tumor biopsies, interacted with host cell components that modulate the evolution parameters of BC. We also examined the EBV replicating form by the titration of serum anti-ZEBRA antibodies. Immunological studies were performed on a series of 35 patients randomly selected from the second half of the survey, involving IFN-γ and TNF-α intracellular immunostaining tests performed *via* flow cytometry analysis in peripheral NK and T cells, in parallel with EBV signature. The effect of the EBV load in the blood or tumor tissue on patient survival was analyzed using univariate and multivariate analyses, combined with an analysis of covariance.

**Results:**

Our study represents the first ever report of the impact of EBV on the clinical outcome of BC patients, regardless of tumor histology or treatment regimen. No correlation was found between: (i) EBV detection in tumor or PBMCs and tumor characteristics; (ii) EBV and other prognostic factors. Notably, patients exhibiting anti-ZEBRA antibodies at high titers experienced poorer overall survival (p = 0.002). Those who recovered from their disease were found to have a measurable EBV DNA load, together with a high frequency of IFN-γ and TNF-α producing PBMCs (p = 0.04), which indicates the existence of a Th1-type polarized immune response in both the tumor and its surrounding tissue.

**Conclusions:**

The replicative form of EBV, as investigated using anti-ZEBRA titers, correlated with poorer outcomes, whereas the latent form of the virus that was measured and quantified using the EBV tumor DNA conferred a survival advantage to BC patients, which could occur through the activation of non-specific anti-tumoral immune responses.

**Electronic supplementary material:**

The online version of this article (doi:10.1186/1471-2407-14-665) contains supplementary material, which is available to authorized users.

## Background

Breast cancer (BC), the most common cancer in women, is considered a heterogeneous disease with pathological characteristics such as morphology, grade, and hormone-receptor profile used in order to stratify tumors into biologically- and clinically-distinct groups [1]. For nearly two decades now, reports have suggested that the Epstein–Barr virus (EBV) [2] may constitute a putative factor in BC natural history [3]. Since 1995, various studies have reported detecting the EBV in BC cases [4–14]. Yet the results remain unconvincing, and their interpretation has been a matter of debate for several years [15–22]. A link between the EBV and BC was first proposed when two studies detected EBV DNA in whole tumor material in 50% of their studied cases [7]. Following this report, other authors detected EBER-2 and LMP-2 DNA by polymerase chain reaction (PCR) in 51% of breast cancers, compared to only 10% in normal tissue from the same patients, thus demonstrating that the EBV could be restricted to tumor epithelial cells [8, 23]. In their study combining laser capture microdissection techniques with real-time quantitative PCR, Arbach *et al.* detected EBV genomes in approximately 50% of BC specimens [4], revealing viral loads which greatly varied from tumor to tumor. Another issue has also been addressed in a previous publication comparing EBV DNA levels in peripheral blood with the viral load in the tumor specimens [14]. Interestingly, the authors of both studies reported finding EBV in the tumor specimens, yet no EBV genomic DNA in peripheral blood, which is consistent with the epithelial localization of the virus. This controversy was later resolved by others, with publications reporting a strict correlation between EBNA-1 expression and EBV DNA detection by PCR [11], although the detection of EBV (protein expression and DNA detection), in terms of it being restricted to tumor epithelial cells, is still a debated issue.

As concerns the impact of the EBV on disease prognosis and evolution, only few studies have clearly addressed the relevant conclusions resulting from various trials [8, 18, 24]. These included, for the most part, contradictory conclusions: (i) some authors demonstrated that the EBV might be associated with aggressive BC forms [4, 6, 8], or may enhance tumorigenic activity [25]; (ii) on the other hand, other studies mentioned the absence of EBV detection in tumor tissue [16–18]; (iii) others demonstrated that the EBV played no relevant role in BC pathogenesis [10].

Here, we have presented prospective data on the effect of EBV infection combined with survival in 85 patients enrolled in a prospective study. Our study aims were concentrated into three axes: i) EBV DNA detection in both BC tissue and peripheral blood mononuclear cells (PBMCs); ii) the IFN-γ and TNF-α intracellular immunostaining test combined with flow cytometry analysis, chosen owing to the fact that cytokines, primarily secreted by activated T cells and natural liller cells, play a crucial role in the response to persistent viral infections [26]; iii) patient clinical outcome and pathological characteristics. Our results demonstrate that the detection of EBV infection, together with immunological studies, could help predict disease outcome in terms of patient survival.

## Methods

### Patients

A total of 85 BC patients were enrolled in the study (Portuguese female patients, primarily at the postmenopausal stage). Their age at diagnosis ranged from 34 to 83 years. The study included only patients diagnosed and treated at the Gynecology Unit of the Coimbra University Hospital, which is the principal general hospital in this area of Portugal, covering a both rural and urban population of approximately 2.3 million people. The size of this population has already been well described in a previous study [27]. BC diagnosis and the histopronostic Scarff-Bloom-Richardson classification (SBR) were conducted using the relevant criteria, as previously described [28]. The treatment protocol for invasive BC was designed in accordance with the 5^th^ National Consensus for Breast Cancer (see Additional file 1). Each patient was classified according to the TNM (tumor-nodes-metastasis) system. The protocol was approved by the medical Ethics Committee of the Coimbra University Hospital (Portugal). The informed consent was obtained from every patient and from every healthy control donor.

### Tumor samples

These were collected prior to chemotherapy or radiotherapy in accordance with the protocols defined in the National Statement on Human Research Involving Humans. On collection, the formalin-fixed and paraffin-embedded tissues were divided into three parts: the first to be submitted for conventional histological study; the second assayed for estrogen and progesterone receptors; the third used in molecular biology assays. Total tumor DNA was extracted from a 10 μm section from each biopsy, as previously described [19].

### Blood samples

Prior to any treatment, 50 ml of whole blood were collected into heparinized tubes from both BC patients and controls (totaling 16 healthy blood donors). Firstly, total peripheral blood mononuclear cells (PBMCs) were isolated by Ficoll density gradient centrifugation (Lymphoprep©, Eurobio France). Enriched PBMCs (1.5 10^7^ cells/0.5 mL) were immediately stored in cryotubes, with 20% DMSO, at -80°C for 48 hours, then frozen in liquid nitrogen until brought out for use. DNA was isolated from the PBMCs by means of the Qiamp DNA blood mini kit (Qiagen, Hilden, Germany) and then quantified. The second step consisted of serum sample collection, with the DNA from 200 μL of serum samples extracted using the same protocol.

### EBV detection by real-time quantitative Light Cycler (LC)-PCR

The amplification and quantification of the EBV DNA were both assessed by real-time PCR on an LC apparatus (Roche Diagnostics), as previously described [29]. An equivalent of 0.5 μg of extracted DNA was used in the PCR. Standard curves for the quantification of EBV DNA were generated using 10-fold serial dilutions of Namalwa cell DNA. In parallel with this, genomic DNA was also quantified for amplification by means of a ribosomal DNA probe/primer set (Eukaryotic 18S rRNA Endogenous Control, Applied Biosystems) as an internal efficiency control. The samples were measured in duplicate. For all samples taken from the 85 patients, the results were given as EBV copy number per μg of total extracted DNA, with a lower detection limit of 5 and 10 copies EBV DNA/μg for PBMCs and tumor biopsies, respectively.

### EBV-related serology

In all patients, EBV serology was determined using two different methods: (i) conventional indirect immunofluorescence assays (IFA) were performed to measure anti-VCA IgGs and anti-early antigens (EA); (ii) we also investigated the reactivation of the lytic cycle through an ELISA titration of the anti-ZEBRA IgGs, as previously described [30, 31]. The results were expressed as optical density (OD) and translated by Pearson’s correlation analysis in order to determine the corresponding anti-ZEBRA antibody titers (1OD ≈ 5000).

### Immunological studies

Immunological studies were performed on the series of 35 patients randomly selected from the second half of the survey. These patients did not differ from the other 50 in terms of diagnostic age, menopausal status, tumor subtype, and EBV DNA load in tumor tissue (p = 0.58) (see Additional file 2: Figure S3). The studies consisted firstly (i) of T and NK cell stimulation, conducted to assess T- and NK-cell ability to produce IFN-γ and TNF-α in response to PMA/ionomycin *in vitro* stimulation. For this, 0.5 mL heparinized blood samples taken from BC patients and female controls were diluted into an equal volume of RPMI 1640 medium. The cells were then stimulated with 50 ng/ml phorbol 12-myristate 13-acetate (PMA; Sigma), and 1 mg/mL ionomycin (Boehringer Mannheim, Germany) in RPMI-1640 medium, containing 10% heat-inactivated fetal calf serum (FCS), 2 mM glutamine, 1% penicillin-streptomycin (Gibco), and 10 *μ*g/mL Brefeldin A (Golgi plug, Sigma, Saint Louis, MO, USA). Unstimulated samples were set up in parallel, but without PMA and ionomycin. Finally, the tubes were incubated for 4 h at 37°C in a humid atmosphere with 5% CO_2_ concentration. The second study (ii) consisted of cellular staining and flow cytometry, including the indirect staining of intracellular cytokines and cell surface molecules, performed throughout according to the manufacturer’s instructions. For a brief description, cells were stained by means of conjugated mAbs PerCP-CD3 and APC-CD56 or APC-CD57 (Pharmingen BDB), directed against T lymphocytes and NK subsets, respectively. The cells were then washed with PBS, fixed, and permeabilized with a Fix & Perm kit. Cells were incubated with anti-IFN-γ-FITC (clone 4S.B3, Pharmingen BDB) and anti-PE-TNF-α (clone Mab11, Pharmingen BDB) antibodies, then washed with PBS, and fixed with 0.5% paraformaldehyde in PBS. The cells (1×10^4^) were analyzed on FACSCalibur flow cytometer using Cell-Quest (BD Biosciences) and Paint-A-Gate 3.0.2 PPC© software (BDB, Coimbra).

### Survival analysis

Our data consisted of overall patient survival (S), defined as the probability that the patient is still alive (S = 1) at a specific time (“t”) during the study period, covering the time of BC clinical diagnosis to the cut-off date of December 2010. During that period, all patients were initially alive (*i.e.,* with S = 1) and may either have gone on to die (therefore S = 0) or stay alive, and may or may not have experienced relapse events, where patients having had surgery suffered from tumor relapse after a disease-free period. The survival analysis was established in order to investigate the effect of EBV infection and other clinicopathological factors on BC patient survival (S). To this end, Cox proportional hazards analyses for S were conducted, applying eight clinicopathological explicative variables or covariates, including: EBV detected in PBMCs (EBV-P) or tumors (EBV-T), relapse, tumor size, lymph node invasion, histological grade (Grade), estrogen/progesterone receptor (ER/PR) status, HER-2 status (HER2), and anti-ZEBRA antibody titration. These were the only eight variables available in the database. The treatment variable was not included in the analyses due to the heterogeneous distribution of treatments with only a very small number of patients in several treatment classes (see Additional file 1).

We proceeded with the following two steps. Step 1: for verification purposes, univariate Cox proportional hazards analyses were performed to calculate patient survival S with each clinicopathological explicative variable X consisting of *S*(*t*) = exp{-*h*(*t*)}, with the hazard function h(t) given by *h*(*t*) = *h*_0_(*t*) × exp{*βX*}, where h_0_(t) is the baseline and β the regression coefficient associated with the variable X. Pearson correlations between all variables were also verified in order to eliminate correlated variables. Step 2: two multivariate Cox models were developed for S with non-correlated clinicopathological variables, excluding and including EBV variables, respectively. For each Cox model, we used the hazard function , where h_0_(t) and β represent the same value, as with the univariate analysis, and the coefficient γ accounts for interactions between variables. All combinations in this hazard function were tested leading to several models, and the model with the lowest AIC (Akaike information criterion) was retained as the best. Following this, the two best models, both excluding and including EBV, were compared for the purposes of assessing the effect of EBV status on patient survival. All statistical analyses were carried out using the free software R Version 2.12.2 (2011-02-25), (Copyright 2011 The R Foundation for Statistical Computing). In R, we applied the functions “coxph” for Cox analyses and “stepAIC” for selecting the best model, according to AIC. Survivor functions were estimated using the Kaplan–Meier method, while hazard ratios or relative risks, given as *RR*_*i*_ = exp(*β*_*i*_) or *RR*_*i*,*j*_ = exp(*γ*_*i*,*j*_), associated with explicative variables were presented with their corresponding 95% confidence intervals (95% CI), and statistical tests were performed at the 5% significance level (p <0.05). In this context, RR >1 corresponds to a negative effect, *i.e.* a decrease in patient survival, while a RR <1 corresponds to a positive effect, *i.e.* an increase in patient survival (“95% CI” stands for the confidence interval at 95%).

## Results

### Global results and EBV status

**Figure 1:**
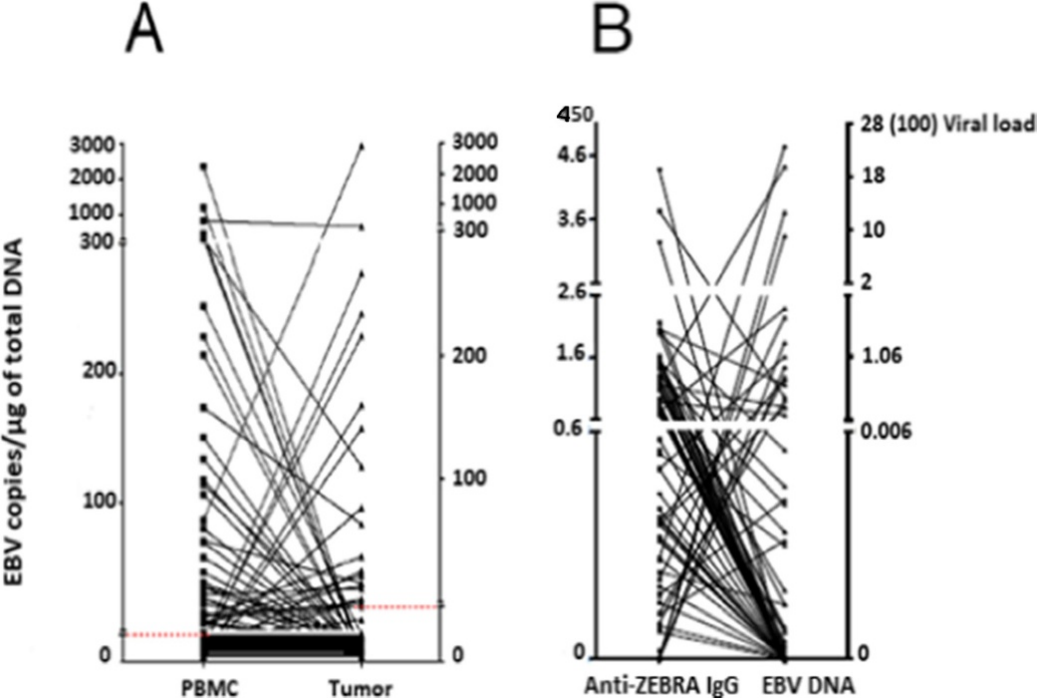
**Quantification of EBV DNA in peripheral blood mononuclear cells (number of copies/μg) from the 85 BC patients.** The detection threshold was 5 and 10 copies EBV DNA/μg for PBMCs and tumor biopsies, respectively. Comparison with EBV load in tumors (number of copies/μg) **(A)** and with anti-ZEBRA antibody titers (in absorbance of 450 nm) **(B)** (an optical density of 1 corresponds to a titer of 1000).

All the patients enrolled in this study were EBV-seropositive (detectable anti-VCA and anti-EBNA IgGs). The histopathologic types of the breast tumors analyzed, as well as patient characteristics, have been summarized in (see Additional file 3: Table S1). In the total 85 whole-blood samples obtained from female BC patients, 40 (47%) were revealed as positive (EBV-P^**+**^ patients) by PCR, with EBV DNA copies ranging from 10 to 2360 copies/μg blood (median: 100 EBV DNA copies/μg). EBV DNA detection was then measured on 85 paraffin-embedded tissues, with 22 out of 85 (25.8%) revealed as positive for EBV (EBV-T^**+**^ patients) and EBV DNA copies ranging from 10 to 2950 copies/μg (median: 84 EBV DNA copies/μg). In comparison, three PBMC samples from 16 healthy control individuals contained EBV DNA (median: 0 copies/μg DNA). None of the 85 tumors in this study had to be excluded due to inadequate control amplification. Given that we examined EBV DNA load in both neoplastic breast tissue and matched peripheral blood, no correlation was found between EBV loads in blood and tumors. It is interesting to note that the viral load was highly variable from tumor to tumor (Figure 1A). The proportion of BC patients negative for EBV DNA in both PBMCs and paraffin embedded tissues (EBV-P^**-**^/EBV-T^**-**^) reached 27%. For a brief overview, no association could be established between EBV load (PBMCs and tumor) and other prognostic factors, including age at diagnosis, tumor size, and lymph node invasion (see Additional file 4: Figures S2A, S2B, S2C, and S2D). Patients without relapse were more likely to exhibit detectable EBV DNA in their blood, as well as EBV to some extent in their tumor. By using the Mann–Whitney test, we revealed that the virus did not seem to have any significant effect on overall survival (p > 0.05), though this result was further tested by means of univariate and multivariate analyses. In another series of experiments exploring reactivating EBV, we investigated the presence of anti-ZEBRA antibodies, which are considered the hallmark of EBV replication activation. As shown in Figure 1B, we observed no association between EBV load in blood and anti-ZEBRA titers. All the patients exhibiting anti-ZEBRA antibodies at high titers (≥5000) had detectable anti-EA IgGs (data not shown).

### Relationship between EBV status and clinical outcome by univariate and multivariate analyses

In order to investigate the effect of EBV load (PBMCs and tumor) on patient survival and its correlation with clinicopathological factors, we conducted both univariate and multivariate analyses in the following manner.

(i) For the univariate analysis of clinicopathological factors, we first observed that some of the variables demonstrated as having a significant effect (p <0.05) on patient survival were, ranked in descending order of importance: “relapse”, “lymph node invasion”, “anti-ZEBRA titration”, and “tumor size” (Table 1 and see Additional file 5: Figure S1). All these variables had a relative risk (RR) >1, corresponding to a negative effect, *i.e.* a decrease in patient survival. Secondly, the Cox univariate analysis revealed that the EBV variables (EBV-P and EBV-T) produced no significant effect on patient survival, which correlated with the preliminary statistical Mann–Whitney test (see above). Finally, the variables of “tumor size” and “lymph node invasion” were found to correlate (r = 0.43, p <0.05), and as a result, the “tumor size” variable was no longer used in the following analyses. In order to determine if the replicating form of EBV had any influence, we verified the impact of the anti-ZEBRA antibody titers on patient survival. Interestingly, patients with high titers of anti-ZEBRA antibodies (≥5000) had a lower overall survival (p = 0.002).

**Table 1:** **Parameters of the univariate analysis of clinicopathological factors**

Explicative variables	Relative risk (95% CI)	p-value
**Tumor size**	2.04 (1.412–2.95)	0.00015
**Grade 1**	-	-
**Grade 2**	1.38 (0.30–6.30)	0.679
**Grade 3**	2.92 (0.63–13.54)	0.171
**Relapse**	14.95 (5.67–39.44)	<0.0001
**EBV-T** ^**+**^	0.795 (0.291–2.173)	0.65
**EBV-P** ^**+**^	1.05 (0.44–2.51)	0.91
**ER/PR positive**	0.48 (0.19–1.20)	0.116
**HER-2 positive**	2.71 (0.795–9.21)	0.111
**Lymph node invasion**	6.32 (2.31–17.3)	0.0003
**Anti-ZEBRA antibody titration**	4,63 (1.75–12.23)	0.002

**Figure 2:**
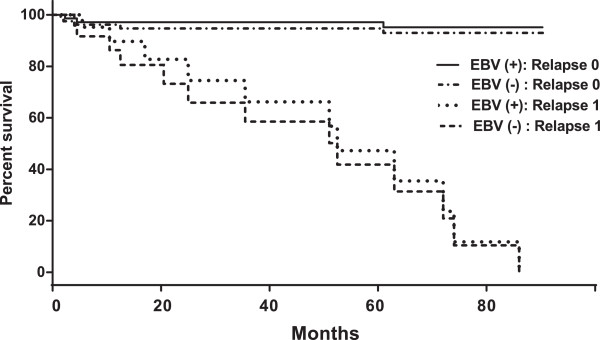
**Relative effect of EBV infection in PBMCs and tumor tissue** ***versus*** **no EBV on survival as a function of time for different relapses.** Relapse 0 means “no relapse”; relapse 1 means “relapse diagnosed”.

**Figure 3:**
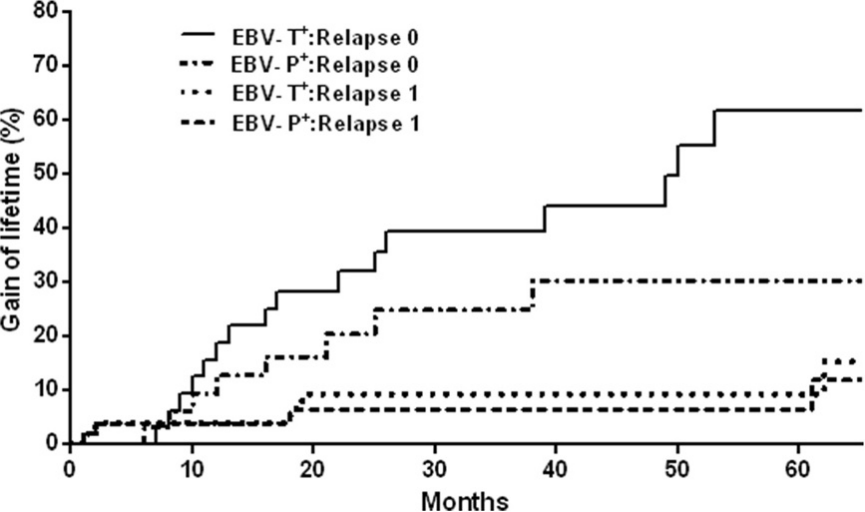
**Effect of EBV-T and EBV-P on the increase in patient survival as a function of time for different relapses.** At 60 months post-diagnosis, the increase in survival is 32% and 8%, respectively, without and with relapse for “EBV-T” patients, *versus* 12% and 6%, respectively without and with relapse for “EBV-P” patients. Relapse 0 means “no relapse”; relapse 1 means “relapse diagnosed”. EBV-T + and EBV-P + represent patients with detectable EBV DNA in tumor tissue and PBMCs, respectively.

EBV: Epstein-Barr virus; T^+^: detected in tumors; P^+^: detected in PBMCs; ER/PR: estrogen/progesterone receptor; HER-2: human epidermal growth factor receptor 2; anti-ZEBRA: antibodies to *Bam*H1 Z Epstein-Barr replication activator.

(ii) To elucidate the possible relationship existing between various clinicopathological factors, we extended our study with the Cox multivariate analysis. Given that the “relapse” variable was revealed to be a function of time, it was applied to stratified data in the Cox analysis. In other words, the baseline of hazard function in Cox models became a function of relapse, as h_0_(t|Relapse). This has been demonstrated in the Kaplan–Meier plot of survival functions as *S*(*t*|Relapse = 0) > *S*(*t*|Relapse = 1). Eventually, we found that the best Cox model for only the hazard function excluding EBV variables involved the “lymph node invasion” and “estrogen/progesterone receptor” variables, with relative risks RR = 5.24 (95% CI = 1.61–17, p = 0.006) and RR = 0.36 (95% CI = 0.13–1, p = 0.05), respectively. The Cox multivariate analysis demonstrated that five clinicopathological variables had a significant effect on patient survival, either independently or in correlation with each other. These consisted of the “lymph node invasion”, “grade”, “HER-2”, and the two EBV variables (EBV-P and EBV-T), of which “lymph node invasion” also appeared significant in the univariate analysis. As presented in Table 2, EBV-T and, to a lesser extent, EBV-P were independent predictive factors for overall survival. When we considered the relationship between EBV status (EBV-T and EBV-P) and the grade, however, we found that the higher the grade, the better the survival for EBV-T^**+**^ patients (RR = 0.0082), as well as for EBV-P^**+**^ patients, yet to a lesser extent (RR = 0.16). A comparison of the two models led to the conclusion that patient survival, when analyzed without EBV variables (h_1_(t) model) and with EBV variables (h_2_(t) model), differed significantly (p = 0.0004). This led us to summarize the multivariate analysis results as follows: (i) For patient survival *vs.* EBV status*,* EBV infection globally improved survival, as EBV-P^**+**^ and EBV-T^**+**^ patients appeared to exhibit higher survival rates than EBV-P^**-**^ and EBV-T^**-**^ patients, regardless of relapses (Figure 2). At 60 months following BC diagnosis, the increase in survival for non-relapsing EBV-positive patients, in comparison with those free of EBV, was approximately 15%, whereas that achieved for relapsing EBV-positive patients, in comparison with those free of EBV, was only 6%; (ii) In terms of EBV-T *vs.* EBV-P effect on patient survival, EBV-T^**+**^ patients exhibited better survival than the EBV-P^**+**^ patients. Figure 3 displays the increase in life expectancy for EBV-T^**+**^ and EBV-P^**+**^ patients, in comparison with those free of EBV. The EBV-T^**+**^ patients presented a 32% and 8% increase in life expectancy, without and with relapse, respectively, in comparison with those free of EBV, while the increase achieved for EBV-P^**+**^ patients was 12% and 6%, respectively.

**Table 2:** **Parameters of the multivariate (Cox model) analysis of clinicopathological factors**

Explicative variables	Relative risk (95% CI)	p-value
**EBV-T** ^**+**^	2.36.10^4^ (152.9-3.7 10^6^)	<0.0001
**Grade**	11.52 (2.28-58.28)	0.003
**EBV-P** ^**+**^	122 (1.5-9.8 10^3^)	0.03
**Lymph node invasion**	16.59 (2.5-110)	0.004
**EBV-T** ^**+**^ **: grade**	0.0082 (0.00071-0.0094)	0.0001
**EBV-P** ^**+**^ **: HER-2**	254 (7.88–8.19 10^3^)	0.002
**EBV-P** ^**+**^ **: grade**	0.16 (0.03-0.96)	0.045

EBV: Epstein-Barr virus; T^+^: detected in tumors; P^+^: detected in PBMCs; HER-2: human epidermal growth factor receptor 2.

The hazard function for the model with EBV variables reads as: _._

The relative risk (RR) >1 corresponds to a negative effect, *i.e.* a decrease in patient survival, while an RR <1 corresponds to a positive effect, *i.e.* an increase in patient survival. “95% CI” stands for the confidence interval at 95%. The variable “Grade” includes all grades.

### Functional evaluation of T/NK cells and clinical outcome

**Figure 4:**
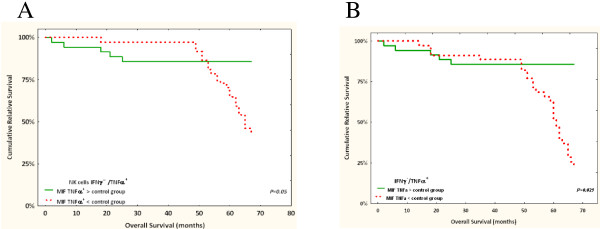
**Clinical outcome of 35 patients with BC (out of all 85 patients) and correlation with frequency of peripheral NK cells and cytokine production (16 healthy individuals enrolled as negative controls).** Overall survival **(A)** in patients with TNF-α expression (MIF) by NK cells > the control group (solid green line) and < the control group (red dashed line). In this group of 35 patients, copies of EBV genomes were detected in PBMCs in 66%, and in tumor tissues in 17%. The clinical outcome of the 35 patients with BC and correlation with TNF- α expression by peripheral T cells. Overall survival **(B)** in patients with TNF-α expression (MIF) by T cells > the control group (solid green line) and < the control group (red dashed).

**Figure 5:**
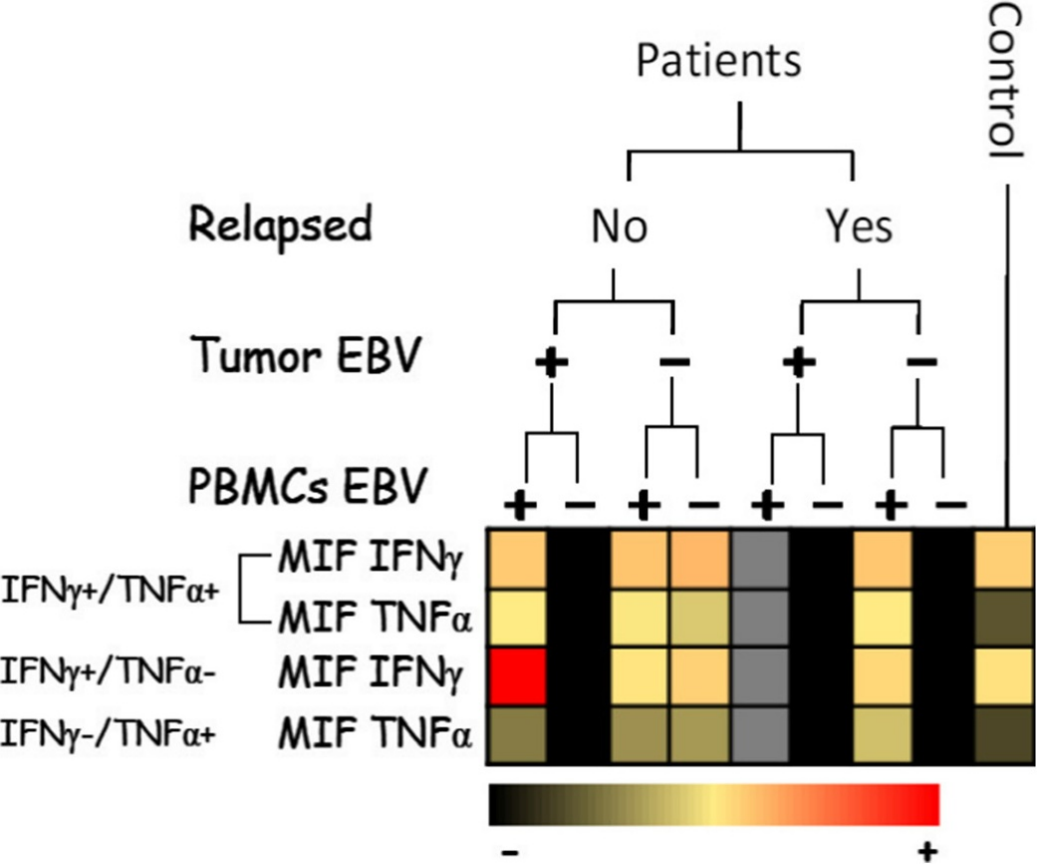
**Synthetic diagram analyzing the impact of the IFN-γ production by peripheral blood mononuclear cells on the clinical outcome according to EBV status in blood and tumor tissue.** PBMC EBV^-^ means <5 EBV DNA copies/μg, PBMC EBV^+^ means >5 EBV DNA copies/μg. Tumor EBV^-^ means <10 EBV DNA copies/μg and tumor EBV^+^ means >10 EBV DNA copies/μg. The intensity of the color is proportional to the amount of cytokine (IFN- γ or TNF-α) production (the black color indicates negative or control basal level, the red color indicates positive or high level).

We assessed the frequency of immunocompetent cells in peripheral blood, as well as TNF-α and IFN-γ production, in order to identify the mechanism by which EBV operates. For EBV status and cumulative survival, the 35 BC patients investigated here were not found to differ from the other 50 (see Additional file 6: Figure S4). Initially, we stimulated the PBMCs with iono/PMA and determined cytokine production as a read-out of cell activation. The IFN-γ production of circulating PBMCs was much greater in EBV-positive patients (EBV-P^+^ or EBV-T^+^) compared to other groups and controls (*p* = 0.04 r = 0.36). In a second step, we investigated both peripheral T-cell and NK-cell response upon PMA/ionomycin activation in BC patients *versus* the control group. Our findings indicated that the frequency of IFN-γ^+^/TNF-α^+^ producing NK cells exhibited the same pattern, with similar mean values (16.2% ± 11) in both relapsing and non-relapsing groups, and regardless of EBV status, with no difference revealed in the control group (15.8% ± 15). We assessed the amount of TNF-α in an IFN-γ^-^/TNF-α^+^ NK cell subset and found it to be higher in non-relapsing patients than in those who underwent relapse. This increase of cytokine production correlated with a higher survival rate (Figure 4A). Nevertheless, we noticed that the non-relapsing patients exhibited an increasing frequency of IFN-γ^+^/TNF-α^+^ NK cells, though a non-significant trend in patient survival (p >0.05) was observed. Following this, we examined the distribution of T lymphocytes along with their capacity to produce TNF-α upon stimulation with PMA/ionomycin. Our findings indicated that TNF-α production was significantly higher in the non-relapsing group than in the relapsing, suggesting a significant correlation between the amount of TNF-α in IFN-γ^**-**^/TNF-α^+^producing T cells and increased survival (*p* = 0.02) (Figure 4B). To investigate further, we divided the patients into two groups: the relapsers and non-relapsers, with each group then further divided according to EBV status (EBV-P^**+**^/EBV-T^**+**^, EBV-P^**+**^/EBV-T^**-**^, EBV-P^**-**^/EBV-T^**+**^, and EBV-P^**-**^/EBV-T^**-**^). We observed that the frequency (%) of PBMCs producing IFN-γ was similar across all groups, with a mean value of 9.1% ± 6.2. The primary difference, however, was focused in the intensity of IFN-γ expression (MIF) (Figure 5). The results indicated that a significant amount of IFN-γ was produced by the peripheral blood mononuclear cells in EBV-P^**+**^/EBV-T^**+**^ patients, with a mean value of IFN-γ expression from T cells calculated as 555 ± 226 *versus* 82 ± 10 in EBV-P^**+**^/ EBV-T^**-**^ patients, and 164 in the single EBV-P^**-**^/ EBV-T^**+**^ patient. As displayed in Figure 5, non-relapsing BC patients with EBV detected in both tumor tissue and PBMCs strongly correlated with a high production of IFN-γ.

## Discussion

A recent literature review evaluated the methodology of all studies published to date, remarking that only four of the 30 studies convincingly demonstrated the presence of EBV in breast tumor tissue [32]. While several research investigations have explored EBV DNA presence in tumor tissue by using Q-PCR [4, 6, 13, 14, 18, 20], their findings have been extremely divergent, with no consensus reached. Nevertheless, it is worth mentioning that the most recent report, published in 2011, found that EBV DNA was present in 33.2% of cases [6]. Another study [4] demonstrated that EBV DNA in breast tumors was detectable when using qPCR in 46% of cases, usually in low copy numbers and heterogeneously distributed. Their results also revealed that the viral load highly varied from tumor to tumor and suggested that EBV infection, at a late stage of tumor development, may enhance its oncogenic properties, such as invasion, angiogenesis, and metastasis. For these reasons, we opted for the qPCR method to investigate the EBV DNA extracted from the whole tumor specimen. With this technique, we uncovered a positive ratio of 25.8% for EBV in tumor tissue (EBV-T^+^ patients) and 47% in peripheral blood (EBV-P^+^). As has been demonstrated by other publications before us [14], the presence of EBV in the tumor specimens coupled with no detection of EBV genomic DNA in the peripheral blood, and *vice-versa*, that we observed are consistent with the epithelial nature of the virus. In this study, we examined both neoplastic breast tissue and matched peripheral blood samples for EBV DNA, in the aims of reporting the impact of EBV on the clinical outcome of BC patients, regardless of tumor histology or treatment regimen. The most prominent results were our ability to demonstrate, through multivariate statistical analysis, that the presence of EBV DNA at any level in both circulating PBMC and tumors was associated with increased lifetime for BC patients. When interpreting the multivariate analysis results, the beneficial role of EBV in the context of BC outcome is particularly striking for patients with high grade tumors, and this effect is all the more impressive for EBV-T^+^ patients, compared to EBV-P^+^ patients. Given that both peripheral and tumor EBV DNA loads were demonstrated to correspond primarily to a latent form of the virus [11, 33–36], we could posit that this latent form, whether tumoral or circulatory, could be beneficial for the patient. In contrast, when verifying the impact of the anti-ZEBRA antibody titers on patient survival, we demonstrated that patients with anti-ZEBRA antibodies at high titers (≥5000) exhibited poorer overall survival (p = 0.002). This observation was in line with other studies investigating other EBV-associated tumors, such as Hodgkin’s Lymphoma (HL) or non-Hodgkin lymphomas [30, 37]. Interestingly, Arbach *et al.* [4] succeeded in detecting ZEBRA transcripts in two of the eight BC biopsy specimens. In BC cases, the expression of ZEBRA could be deleterious, namely because ZEBRA is able to induce metalloproteinase expression that may contribute to invasion and metastasis [38]. Two other interesting features were: (i) the absence of correlation between EBV peripheral load and anti-ZEBRA antibodies; (ii) the absence of correlation between EBV load in peripheral blood and tumor biopsies. These figures could therefore demonstrate a different pattern of EBV, in the context of BC, compared to other EBV-positive cancers originating in the epithelial cells [30, 39–41]. All in all, these findings suggest a compartmentation between the breast tumor area and periphery. In this study, we uncovered a new significance of the peripheral and tumoral global EBV load, which appeared to represent not only a harmless passenger, as suggested by others [12], but also a contributory function to the body’s immune reaction against the tumor. Interestingly, a recent report noted that the EBV may contribute to the risk of BC, and that this contribution may be modified by genetic variations in IFN-γ [42]. In our study, the most critical results were the frequency of interferon-γ producing PBMCs in non-relapsing patients with detectable EBV in blood and tumor tissue. Another critical finding was the greater quantities of TNF-α in the NK cells and T lymphocytes of patients with favorable outcome, as demonstrated by the survival curves. This suggests that these cells are engaged in a Th1-oriented immune activation process, creating an anti-tumoral response in a non-specific manner, with the EBV possibly playing a facilitating role in this response. It appeared possible that the presence of EBV, in tissue and the periphery, stimulated the host immune response, boosting both IFN-γ and TNF-α levels, leading to a favorable outcome in these patients. Nevertheless, the role of the EBV in both blood and tumors in this immune stimulation remained unclear. Taking into account the multivariate analysis and model comparison approach, therefore, and considering the clinical outcome, we could speculate that the role of the EBV is more pronounced in tumor tissue than in peripheral blood. In contrast to what has previously been described, this study may postulate that the EBV, in its latent form, can act as a co-factor for the anti-tumoral immune response. It is worth mentioning similar results obtained recently in Hodgkin’s disease (HD) cases, concerning another EBV-related tumor [43]. In this context, other authors analyzing classical Hodgkin’s lymphoma tissue found that the outcome of the patients may be related to the tumor microenvironment, which in turn may be influenced by EBV infection, suggesting that the EBV could favor a Th1-type immune response in a non-specific way, demonstrating improved outcomes for EBV-positive patients compared to their EBV-negative counterparts [44, 45]. It is interesting to note that this effect has been reported as being age-dependent in the case of EBV-positive Hodgkin’s lymphoma [46]. Nevertheless, our population-based study has led us to conclude that this effect exists regardless of age stratification.

The increased immune response exhibited by EBV-T^+^/EBV-P^+^ patients could result from a cooperation between epithelial cells, dendritic cells, NK cells, and B or T lymphocytes [47]. The frequency of T and NK cells producing IFN-γ, as well as the cytokine quantity at a single cell level observed in EBV-T^+^/EBV-P^+^ patients, indicates that the EBV, in its latent form, induces a prolonged state of anti-tumor immune reaction. The durability of this reaction led us to hypothesize that EBV infection represents a truly symbiotic relationship, by means of heightened innate immune activation, as was recently demonstrated with other herpes viruses [48]. Accumulating evidence has indicated that all three herpes virus subfamilies in latent forms in humans involved chronic, low-level immune activation accompanied by IFN-γ and TNF-α secretion in response to frequent yet subclinical viral reactivation [49–51]. The plausible hypothesis to explain this immune activation could also involve either the chronic presentation of viral antigens or *trans*-activation of HERV-K [52, 53], or both, resulting in prolonged T–cell activation and IFN-γ secretion. This is likely given that HERV surface envelope proteins have been demonstrated to provide target antigens recognizable by cytotoxic T-cells, antibodies [53], or dendritic cells, with the capacity to support a Th1-like process of Th cell differentiation [54]. The negative confounding and apparent link between tumor grade and favorable outcome for EBV-T^**+**^ patients could be accounted for by this putative viral cross-talk, as previous studies have demonstrated that the higher the tumor grade, the greater the expression of tumor HERV [55].

## Conclusion

Our findings have revealed the following unexpected properties of this so-called “double faceted” EBV: (i) the latent form of this virus, measured and quantified by the tumor viral EBV DNA, confers a survival advantage to BC patients; (ii) there is an association between high anti-ZEBRA titers and poor outcome, though the high anti-ZEBRA response could be the result of late stage cancer and not the cause of poor outcome. Given that this study assessing the beneficial effects of the EBV was conducted over a long time period, these results are a relevant basis for future studies involving a larger patient population.

## Electronic supplementary material

Additional file 1: **Description of the treatment protocols.** (DOC 28 KB)

Additional file 2: Figure S3: Diagram comparing the EBV status in the two patient groups (35 *versus* 50 patients). Patient EBV characteristics for each group were not statistically different (p = 0.58). (DOC 49 KB)

Additional file 3: Table S1: Characteristics of patients (n = 85) and tumors (DI = Ductal invasive Carcinoma, DIS = Ductal *in situ* carcinoma, LI = Lobular Invasive carcinoma), clinical outcome, detection of Epstein-Barr-Virus DNA by PCR in Peripheral Blood Mononuclear Cells and tumor samples. Mann–Whitney U test was used for determine differences between groups. p-value < 0.05 was considered statistically significant. The primary end point was disease-free survival which was defined by the time interval between the diagnosis of the disease and the date of relapse or death (any cause). Overall survival was defined by the time between the diagnosis and the date of death (any cause). (DOC 38 KB)

Additional file 4: Figure S2: Correlation between EBV status and clinical patient outcome: the first set (S2A, S2B, and S2C) illustrates an absence of correlation between EBV status and the clinical outcome of patients without (A and B) and with metastatic lymph nodes (C). Figure S2D illustrates an absence of correlation between EBV status and clinical patient outcome in terms of tumor size (pT >2). In all cases, overall survival was defined in the Methods section. (DOC 454 KB)

Additional file 5: Figure S1: Effect of relapse on overall patient survival as a function of time. (DOC 32 KB)

Additional file 6: Figure S4: Comparison of EBV status in the two patient groups (35 *versus* 50 patients) in terms of clinical outcome. (DOC 32 KB)

## Abbreviations

AIC: Akaike information criterion
Anti-IFN-γ-FITC: Monoclonal antibody to IFN-γ fluorescein isothiocyanate conjugated
anti-VCA: Antibodies to viral-capsid antigen
anti-ZEBRA: Antibodies to *Bam*H1 Z Epstein-Barr replication activator
APC-CD56 or APC-CD57: Monoclonal antibody to CD56 or CD57 allophycocyanin conjugated
BC: Breast cancer
BDB: Becton Dickinson Biosciences
coxph: Cox analyses
DMSO: Dimethyl sulfoxide
DNA: Deoxyribonucleic acid
EA: Epstein Barr virus early antigens
EBV: Epstein Barr virus
EBV-P: EBV detected in PBMCs
EBV-T: EBV detected in tumors
ER/PR: Estrogen/progesterone receptor
EBNA-1: Epstein–Barr nuclear antigen 1
EBER-2: Epstein-Barr virus (EBV)-encoded RNA 2
FCS: Fetal calf serum
HD: Hodgkin’s disease
HER-2: Human epidermal growth factor receptor 2
HERV-K: Human endogenous retrovirus-K
IFA: Indirect immunofluorescence assay
IFN-γ: Interferon γ
LMP-2: EBV latent membrane protein 2
NK cells: Natural killer cells
qPCR: Quantitative polymerase chain reaction
PBMCs: Peripheral blood mononuclear cells
PE-TNF-α: Monoclonal antibody to TNF-α phycoerythrin (PE) conjugated
PerCP-CD3: Monoclonal antibody to CD3 peridinin-chlorophyll proteins conjugated
Paint-A-Gate 3.0.2 PPC©: Software program, Becton Dickinson Biosciences
PMA: Phorbol 12-myristate 13-acetate
RR: Relative risk
S: Overall patient survival
SBR: Scarff-Bloom-Richardson classification
stepAIC: Selection of the best model according to AIC
TNF-α: Tumor necrosis factor α
ZEBRA: *Bam*H1 Z Epstein-Barr replication activator.
