# Supplementary material for: Epstein-Barr virus infection and clinical outcome in breast cancer patients correlate with immune cell TNF-α/IFN-γ response
Source: BMC Cancer. 2014 Sep 11;14:665. doi: 10.1186/1471-2407-14-665 (PMC4171567; doi:10.1186/1471-2407-14-665)
Supplement: Supplementary file 1 — Additional file 1: Description of the treatment protocols. (DOC 28 KB) [file 12885_2014_4850_MOESM1_ESM.doc]

**Additional file 1**

**Description of the treatment protocols**

In total, 85 patients with breast carcinoma (BC) were enrolled in the study (Portuguese female patients, primarily at postmenopausal stage). Their age at diagnosis ranged from 34 to 83 years. All the included patients were diagnosed and treated in the Gynecology Unit of the Coimbra University Hospital, the principal general hospital in this area of Portugal covering both rural and urban populations totalling approximately 2.3 million people. The size of the target population was 200,000 (Coimbra,-Portugal), which is significant when compared to that of Stockholm (Sweden) (164,000) or Turin (Italy) (73,000). Most BC patients (64.6%) were postmenopausal. The treatment protocol for breast invasive cancer followed by the service was in accordance with the 5th National Consensus of Breast Cancer, as per the last revision from April 2005. Following establishment of histological diagnosis of invasive breast cancer, the patients were classified according to the TNM (tumor-nodes-metastasis) staging system. The decision to initiate any treatment such as surgery or chemotherapy depended on the size of the injuries, presence or absence of axillary adenopathy, and hormonal status of the patient. For all tumors <5cm without concrete axillary adenopathy, the initial treatment was surgical. In premenopausal patients with tumors >2cm (pT>2) or positive axillary adenopathy, the initial treatment was neo-adjuvant chemotherapy (see below). For local advanced tumors, characterized by a size >5cm, fixed axillary adenopathy or invasion of the skin/thoracic wall, along with the presence of inflammatory carcinoma, the initial treatment was always neoadjuvant chemotherapy, with surgery performed at a later time. Neo-adjuvant chemotherapy was also considered for reducing tumor size and enabling conservative surgery. The surgical treatment consisted of conservative surgery, including widened tumor extraction or quadrantectomy, associated with homolateral axillary draining, along with modified radical mastectomy or simple mastectomy. Conservative surgery was carried out for tumors <3cm. The technique of the sentinel lymph node was considered for women with no palpable axillary lymph nodes on clinical examination. Whenever possible, patients were offered the possibility of undergoing reconstructive surgery, either immediately or deferred. Radiotherapy was performed, following surgery, in all conservative surgery cases when the number of positive axillary lymph nodes was >3, as well as in all local advanced breast cancer cases.

Approximately eight different treatment protocols were used, with the following distribution: 33 of the total 85 patients were treated with adjuvant chemotherapy, consisting of 22 with FEC100, two with FEC100 +T, one with CMF, one with CMF + AT, one with ET + AGOC, two with EC, and four with EC+T. A total of eight patients were treated with hormone therapy only, five radiotherapy only, six chemotherapy + hormone therapy, 13 chemotherapy + radiotherapy, 11 radiotherapy + hormone therapy, and nine chemotherapy + hormonotherapy + radiotherapy. The remaining 37 patients received adjuvant hormone therapy with tamoxifen, goserelin, exemestane, or anastrozole.
