# Supplementary figures and images for: Epstein-Barr virus infection and clinical outcome in breast cancer patients correlate with immune cell TNF-α/IFN-γ response
Source: BMC Cancer. 2014 Sep 11;14:665. doi: 10.1186/1471-2407-14-665 (PMC4171567; doi:10.1186/1471-2407-14-665)

**Additional file 2: Figure S3**


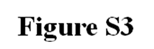

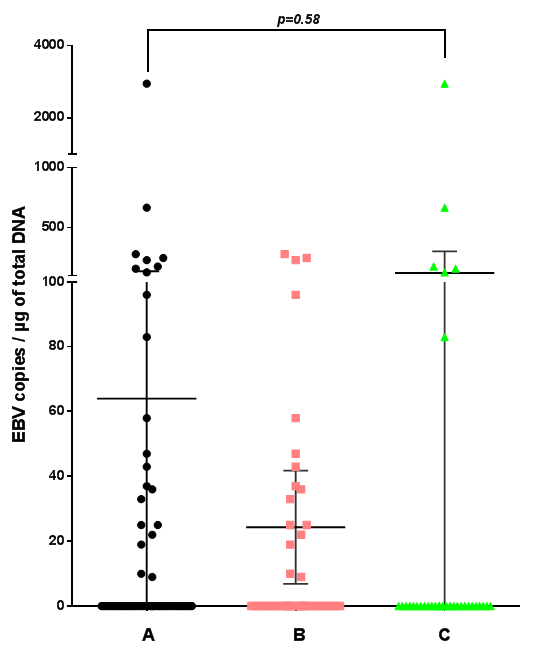

Supplement: Supplementary file 2 — Additional file 2: Figure S3: Diagram comparing the EBV status in the two patient groups (35 versus 50 patients). Patient EBV characteristics for each group were not statistically different (p = 0.58). (DOC 49 KB) [file 12885_2014_4850_MOESM2_ESM.doc]

**Additional file 4: Figure S2**


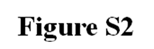

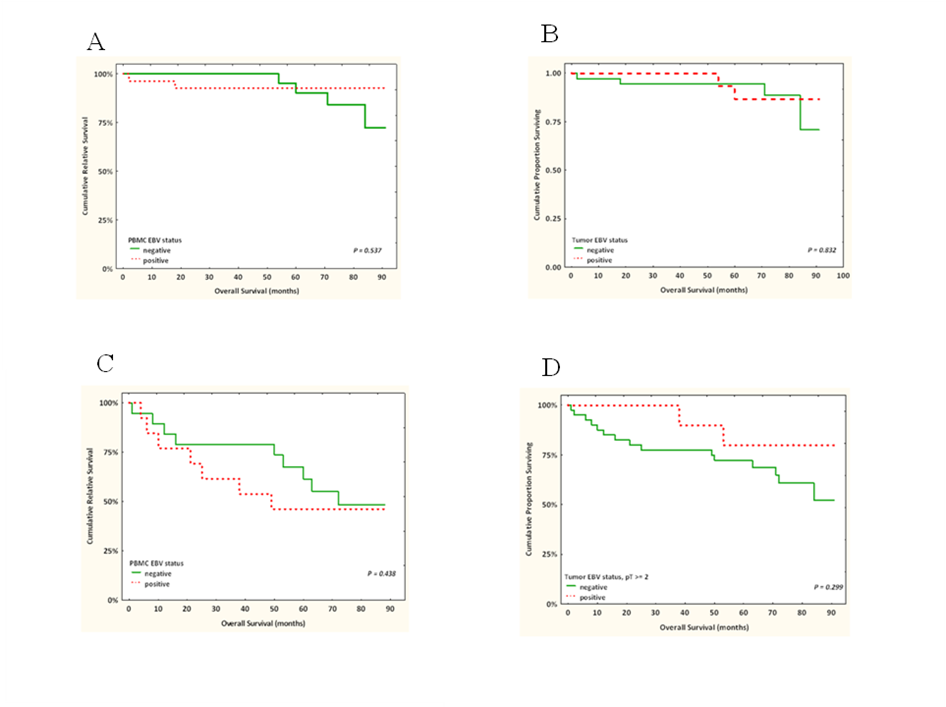

Supplement: Supplementary file 4 — Additional file 4: Figure S2: Correlation between EBV status and clinical patient outcome: the first set (S2A, S2B, and S2C) illustrates an absence of correlation between EBV status and the clinical outcome of patients without (A and B) and with metastatic lymph nodes (C). Figure S2D illustrates an absence of correlation between EBV status and clinical patient outcome in terms of tumor size (pT >2). In all cases, overall survival was defined in the Methods section. (DOC 454 KB) [file 12885_2014_4850_MOESM4_ESM.doc]

**Additional file 5: Figure S1**


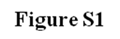

Supplement: Supplementary file 5 — Additional file 5: Figure S1: Effect of relapse on overall patient survival as a function of time. (DOC 32 KB) [file 12885_2014_4850_MOESM5_ESM.doc]

**Additional file 6: Figure S4**


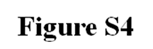

Supplement: Supplementary file 6 — Additional file 6: Figure S4: Comparison of EBV status in the two patient groups (35 versus 50 patients) in terms of clinical outcome. (DOC 32 KB) [file 12885_2014_4850_MOESM6_ESM.doc]
