# Supplementary material for: Epstein-Barr virus infection and clinical outcome in breast cancer patients correlate with immune cell TNF-α/IFN-γ response
Source: BMC Cancer. 2014 Sep 11;14:665. doi: 10.1186/1471-2407-14-665 (PMC4171567; doi:10.1186/1471-2407-14-665)
Supplement: Supplementary file 3 — Additional file 3: Table S1: Characteristics of patients (n = 85) and tumors (DI = Ductal invasive Carcinoma, DIS = Ductal in situ carcinoma, LI = Lobular Invasive carcinoma), clinical outcome, detection of Epstein-Barr-Virus DNA by PCR in Peripheral Blood Mononuclear Cells and tumor samples. Mann–Whitney U test was used for determine differences between groups. p-value < 0.05 was considered statistically significant. The primary end point was disease-free survival which was defined by the time interval between the diagnosis of the disease and the date of relapse or death (any cause). Overall survival was defined by the time between the diagnosis and the date of death (any cause). (DOC 38 KB) [file 12885_2014_4850_MOESM3_ESM.doc]

**Additional file 3: Table S1**

| **Characteristics** | **All patients** | | **Disease Free Interval** | | **Overall Survival** | |
| --- | --- | --- | --- | --- | --- | --- |
|  | **Status** | **Nr** | **%** | **p** | **%** | **p** |
| **Lymph node invasion** | No  Yes | 53  32 | 88.7  56.3 | <0.001 | 88.7  50 | <0.001 |
| **ER/PR** | Negative  Positive | 21  64 | 64.7  81.3 | 0.129 | 58.8  78.1 | 0.108 |
| **HER-2 status** | Negative  Positive | 79  6 | 77.2  66.7 | 0.418 | 75.9  50 | 0.096 |
| **Tumor histology** | DI  DIS  LI | 72  9  4 | 75  77.8  100 | 0.536 | 73.6  77.8  75 | 0.949 |
| **Tumor size (pT)** | 1  2  3  4 | 35  34  3  13 | 91.4  76.5  100  30.8 | <0.001 | 85.7  79.4  100  23.1 | <0.001 |
| **Grade** | I  II  III | 12  48  25 | 91.7  77.1  68 | 0.192 | 83.3  77.1  64 | 0.218 |
| **EBV status** | PBMCs  EBV-P^-^  EBV-P^+^ | 45  40 | 73.3  80 | 0.584 | 71.1  77.5 | 0.818 |
|  | Tumor  EBV-T^-^  EBV-T^+^ | 63  22 | 74.6  81.6 | 0.504 | 74.6  72.7 | 0.939 |
